# Proteome-wide mendelian randomization investigates potential associations in heart failure and its etiology: emphasis on PCSK9

**DOI:** 10.1186/s12920-024-01826-6

**Published:** 2024-02-21

**Authors:** Lichao Lin, Huizhen Yu, Yan Xue, Liman Wang, Pengli Zhu

**Affiliations:** 1 Shengli Clinical Medical College of Fujian Medical University, Fuzhou, People’s Republic of China; 2 Department of Geriatric Medicine, Fujian Provincial Hospital, Fuzhou, People’s Republic of China; 3 Fujian Provincial Key Laboratory of Geriatrics, Fuzhou, People’s Republic of China; 4 Fujian Provincial Center for Geriatrics, Fuzhou, People’s Republic of China; 5 Department of Cardiology in South Branch, Fujian Provincial Hospital, Fuzhou, People’s Republic of China; 6 Department of Pharmacy in South Branch, Fujian Provincial Hospital, Fuzhou, People’s Republic of China

**Keywords:** Heart failure, Coronary heart disease, Mendelian randomization, Circulating proteins, PCSK9

## Abstract

**Background:**

Heart failure (HF) is a prevalent clinical syndrome with diverse etiologies. It is crucial to identify novel therapeutic targets based on underlying causes. Here, we aimed to use proteome-wide Mendelian randomization (MR) analyses to identify the associations between genetically predicted elevated levels of circulating proteins and distinct HF outcomes, along with specific HF etiologies.

**Methods:**

Protein quantitative trait loci (pQTL) data for circulating proteins were sourced from the Atherosclerosis Risk in Communities (ARIC) study, encompassing 7,213 individuals and profiling 4,657 circulating proteins. Genetic associations for outcomes were obtained from the HERMES Consortium and the FinnGen Consortium. Colocalization analysis was employed to assess the impact of linkage disequilibrium on discovered relationships. For replication, two-sample MR was conducted utilizing independent pQTL data from the deCODE study. Multivariable MR (MVMR) and two-step MR were further conducted to investigate potential mediators.

**Results:**

Two proteins (PCSK9 and AIDA) exhibited associations with HF in patients with coronary heart disease (CHD), and four proteins (PCSK9, SWAP70, NCF1, and RELT) were related with HF in patients receiving antihypertensive medication. Among these associations, strong evidence from subsequent analyses supported the positive relationship between genetically predicted PCSK9 levels and the risk of HF in the context of CHD. Notably, MVMR analysis revealed that CHD and LDL-C did not exert a complete mediating effect in this relationship. Moreover, two-step MR results yielded valuable insights into the potential mediating proportions of CHD or LDL-C in this relationship.

**Conclusions:**

Our findings provide robust evidence supporting the association between PCSK9 and concomitant HF and CHD. This association is partly elucidated by the influence of CHD or LDL-C, underscoring the imperative for additional validation of this connection and a thorough exploration of the mechanisms through which PCSK9 directly impacts ischemic HF.

**Supplementary Information:**

The online version contains supplementary material available at 10.1186/s12920-024-01826-6.

## Background

Heart failure (HF) is a major contributor to global morbidity and mortality, posing a significant public health burden [1]. This complex syndrome derived from diverse etiologies, with coronary heart disease (CHD) and hypertension being the predominant culprits [2]. Despite efforts towards primary prevention by addressing established risk factors, the incidence of HF continues to escalate and the prognosis remains unfavorable [3, 4]. Consequently, there is a pressing need to identify novel therapeutic targets for HF [5, 6].

Protein convertase subtilisin/kexin type 9 (PCSK9) is a crucial enzyme that maintains lipid metabolism balance by targeting hepatic low-density lipoprotein receptor (LDLR), leading to the development of PCSK9 inhibitors [7–9]. Alirocumab and Evolocumab are clinically applied PCSK9 inhibitors known for their effective lipid-lowering effects and reliable safety profiles [10, 11]. However, the potential of PCSK9 as an intervention target for HF remains uncertain. In the BIOSTAT-CHF cohort, it was observed that circulating PCSK9 levels were notably elevated in HF patients, correlating positive correlation with the risk of mortality in this population [12]. Additionally, other observational studies have independently established a link between circulating PCSK9 levels and a diminished left ventricular ejection fraction (LVEF) following myocardial infarction (MI) [13, 14]. These findings suggest the potential of PCSK9 as a viable therapeutic target for HF. Conversely, another observational study found no association between PCSK9 and HF after adjusting for confounding factors [15]. The conflicting clinical finding raised concerns about the use of PCSK9 inhibitors in HF [16]. This concern was substantiated by a previous meta-analysis based on randomized controlled trials (RCTs), demonstrating that while PCSK9 inhibitors reduce the risk of major adverse cardiovascular events (MACE), non-fatal MI, and stroke, they do not significantly affect HF [17]. Moreover, the application of Evolocumab may increase the risk of all-cause mortality (OR: 1.12; 95% CI: 1.00-1.25) [18]. Furthermore, a recent RCT study indicated that Alirocumab reduces MACE in patients without HF but not in those with HF [19]. Currently, there is only one clinical trial (Phase II) investigating the role of Evolocumab in ischemic HF with reduced ejection fraction (HFrEF) (ClinicalTrials.gov Identifier: NCT03791593). Due to the potential adverse risk, more robust evidence establishing causality between PCSK9 and HF is imperative before conducting larger clinical trials.

Mendelian randomization (MR) analysis is a strategy that employs genetic variation as instrumental variables (IVs) to assess the potential causal relationship between exposure and outcome [20–22]. Leveraging genotypes that are not susceptible to confounding factors or reverse causality, MR holds the potential to provide unbiased estimates of causality [23]. Several MR studies have provided support for a genetically predicted link between PCSK9 and HF [24–26]. However, another MR study failed to establish such a relationship in HF patients experiencing non-ischemic cardiovascular disease (CVD) events [27]. Based on these results, we propose the hypothesis that the association of PCSK9 may differ between ischemic and non-ischemic HF. Notably, these MR studies were predominantly based on data from Genome-Wide Association Study (GWAS) on lipid traits or expression quantitative trait loci (eQTL) about PCSK9. Nevertheless, these data could not comprehensively capture the underlying biological mechanisms, particularly those involving horizontal pleiotropy [28]. Horizontal pleiotropy refers to pathways where the effects of variants on diseases occur independently of their causal influence, such as through alternative splicing or micro-RNA effects [28]. Therefore, it is necessary to validate our hypothesis using protein quantitative trait loci (pQTL) data.

In this study, we employed pQTL data to identify potential therapeutic targets for diverse HF outcomes and specific HF etiologies. In the discovery stage, our proteome-wide MR analysis unveiled associations linking circulating PCSK9 with HF in patients with CHD, as well as HF in patients receiving antihypertensive medication. Notably, no such association was observed for all-cause HF. Supplementary proteome-wide MR analyses affirmed the potential causal connection between circulating PCSK9 and ischemic heart disease, whereas no statistically significant association emerged in essential hypertension. In the replication stage, we validated the association between PCSK9 and HF in patients with CHD. Furthermore, we employed multivariable MR and two-step MR analyses to gain insights into potential mediators in this association. The findings of our study lay the groundwork for preclinical investigations on PCSK9 and contribute to the design of future clinical trials involving PCSK9 inhibitors for ischemic HF.

## Methods

### Study design and ethical approval

**Fig. 1:**
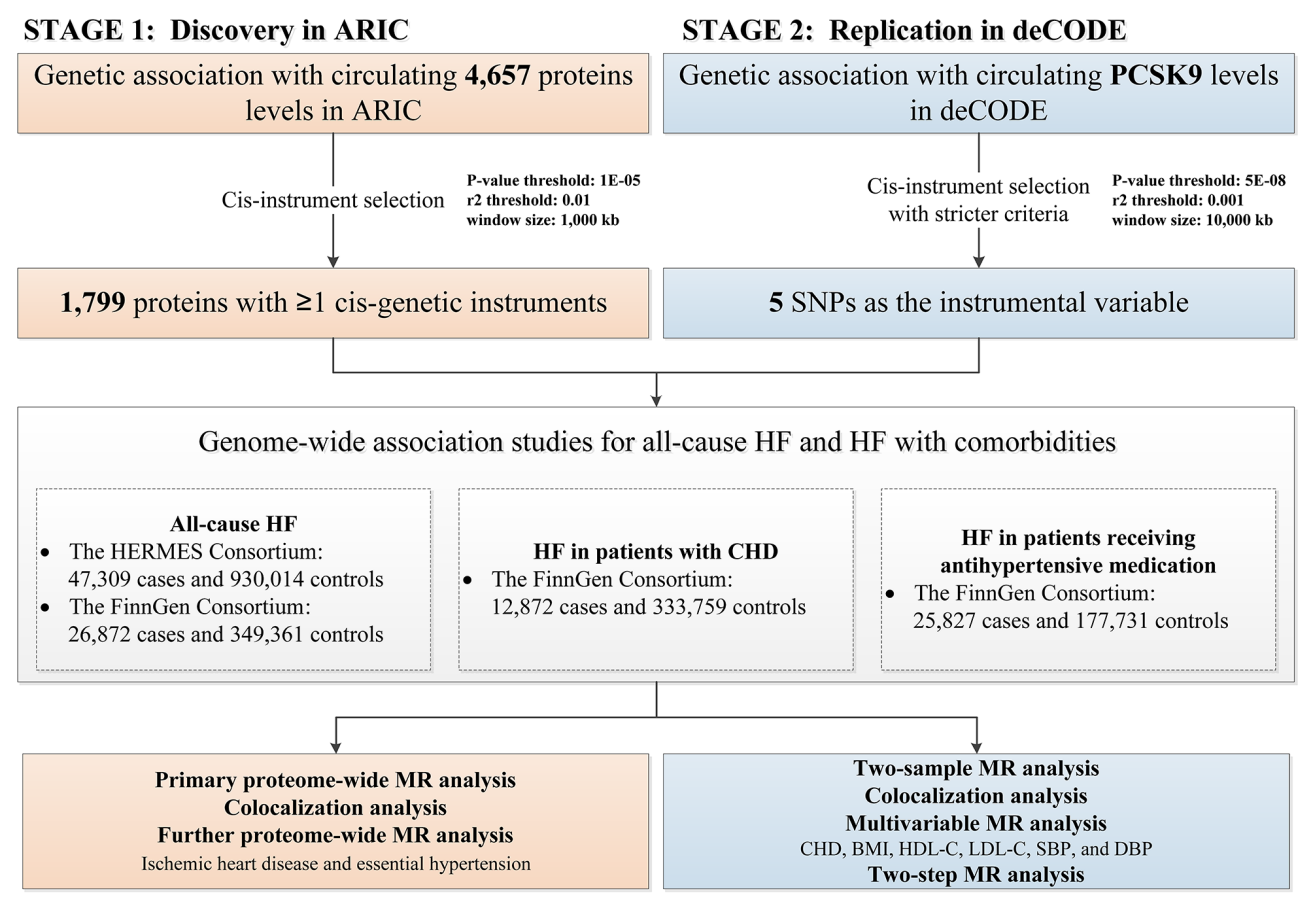
Flowchart of the Study design. ARIC, Atherosclerosis Risk in Communitiess study; deCODE, Diabetes epidemiology: collaborative analysis of diagnostic criteria in Europe; SNP: single nucleotide polymorphism; HF: heart failure; CHD: Coronary heart disease; BMI: Body mass index; HDL-C: High density lipoprotein cholesterol; LDL-C: Low density lipoprotein cholesterol; SBP: Systolic blood pressure; DBP: Diastolic blood pressure

The overview of our study is depicted in Fig. 1, which outlines the key elements and procedures employed. In line with best practices, our study adhered to the Strengthening the Reporting of Observational Studies in Epidemiology using Mendelian Randomization (STROBE-MR) checklist (Table S1) [29]. The pQTL GWAS summary statistics data employed in this paper were sourced from the publicly accessible ARIC study and the deCODE study. (Table S2). The remaining GWAS summary statistics data were sourced from other publicly available websites, and they do not contain any personal identifying information of participants (Table S2). Therefore, no additional ethical approval was required for the present study.

### Proteomic data sources

In the discovery stage, the pQTLs associated with 4,657 circulating proteins were extracted from the Atherosclerosis Risk in Communities (ARIC) study, which included 7,213 individuals of European American descent [30]. Supplementary details regarding data measurement, adjustment for potential confounders, and other processing steps applied to the ARIC study are available in the original publication (Table S2). In our study, the analysis encompassed all 4,657 cis-pQTLs associated with circulating proteins.

In the replication stage, the pQTL associated with circulating PCSK9 was obtained from an independent large-scale pQTL study conducted in 35,559 individuals of Icelandic descent, which was part of the deCODE study [31]. Supplementary information on the data measurement and processing of the deCODE study can be found in the original publication (Table S2). In our study, we extracted all genetic variants associated with PCSK9 and SWAP70, supplementing any missing SNP information with data from the 1,000 Genomes project (GRCh38).

### Outcome data sources

The primary outcomes investigated in our study encompassed all-cause heart failure (HF), HF in patients with coronary heart disease (CHD), and HF in patients receiving antihypertensive medication. The GWAS summary statistics data for these specific HF outcomes were derived from two prominent consortia: the HERMES Consortium and the FinnGen Consortium. The HERMES study consisted of a substantial sample size of 47,309 HF cases and 930,014 controls across 26 cohorts, all of European ancestry. The HF cases in this study represented diverse etiologies. Genetic associations were adjusted for age, sex, and genetic principal components. In the FinnGen study, the phenocodes “I9_HEARTFAIL_ALLCAUSE,” “I9_HEARTFAIL_AND_CHD,” and “I9_HEARTFAIL_AND_ANTIHYPERT” correspond to all-cause HF with 26,872 cases and 349,361 controls, HF in patients with CHD with 12,872 cases and 333,759 controls, and HF in patients receiving antihypertensive medication with 25,827 cases and 177,731 controls, respectively (Version R9). All participants in both consortia were of European ancestry, and there was no overlap between the HERMES Consortium and the FinnGen Consortium.

Significantly, there existed overlap of cases in these HF outcomes from the FinnGen study, reflecting the complex etiology of HF patients. Comprehensive information, including exclusion criteria, sex distribution, and mean age at the first event for each outcome, were available in Table S3. Additionally, to assess the robustness of the identified PCSK9-outcomes association in the HF outcomes, we conducted proteome-wide MR analyses for ischemic heart disease (IHD) and essential hypertension as supplementary investigations. Detailed information on these outcomes can be found in Table S2.

### Selection of genetic instruments

Cis-pQTLs associated with 4,657 circulating proteins were extracted from the ARIC study. Given that the cis-region of a gene represents a small portion of the genome, we relaxed the conventional MR significance threshold to *P* < 1E-05. Instrumental variables (IVs) were selected based on a linkage disequilibrium (LD) *r*^2^ threshold of 0.01 and a window size of 1,000 kb. As a result, 1,799 proteins were chosen for MR analysis. These proteins were then matched with outcomes, excluding proteins that lacked SNPs in the outcomes, resulting in 1,773 proteins from the HERMES study and 1,767 proteins from the FinnGen study for the discovery analyses.

For replication, we obtained comprehensive GWAS data on the circulating PCSK9 protein levels from the deCODE study. To ensure consistency with the ARIC study, we extracted cis-pQTLs for PCSK9 within a +/−500 kb of the transcription start site (TSS) range. This approach was adopted since cis-pQTLs are more prone to exhibit protein-specific effects [32]. Subsequently, we employed stricter criteria (*P* < 5E-08, r^2^ threshold = 0.001, window size = 10,000 kb, and European 1,000 Genome Project as the reference panel) to identify SNPs associated with circulating PCSK9 protein levels as IVs. Multivariable MR and two-step MR also adopted the stricter criterion for IVs screening.

These instrumental variables (IVs) for MR had to follow several rules: (1) IVs must be strongly correlated with the exposure variable; (2) IVs should not be related to potential confounding factors; (3) IVs can only affect the outcome variable through the exposure variable. We then calculated the proportion of variance explained and F-statistic for each SNP to assess their strength as IVs. The strength of each instrument was assessed by computing the F-statistic with the formula: F = R^2^⋅(N − k−1)/((1 − R^2^)⋅k), where R^2^ represents the proportion of variability in physical activity explained by each instrument, N denotes the sample size of the GWAS for the SNP-outcomes association, and k represents the number of instrumental variables [33]. Generally, an F-statistic greater than 10 is considered robust enough to mitigate the influence of potential bias [34]. Detailed information on the explained variance and F-statistic for each SNP can be found in Table S4-5.

### Discovery in ARIC

Proteome-wide MR approach was conducted to identify potential therapeutic targets for various heart failure (HF) outcomes. For proteins with only one single nucleotide polymorphism (SNP) available as the genetic instrument, the Wald ratio was calculated. For proteins with two or more SNPs as genetic instruments, the inverse variance weighted (IVW) was performed as the primary method [35]. In this study, we employed the Bonferroni correction for multiple testing, implementing an allowable type I error rate (α) of 0.05/N, where N indicates the number of outcomes tested under study. Supplementary methods, including weighted median and MR-Egger, were employed for proteins with three or more SNPs as genetic instruments. Other sensitivity analyses, such as MR- Egger regression intercept analysis and Cochran’s Q statistical analysis, were performed to evaluate horizontal pleiotropy and heterogeneity, ensuring the robustness of the results [36–38].

Colocalization analysis was utilized to further validate the findings. The colocalization method primarily assessed the validity of the instrumental variable hypothesis, taking into account whether the potential genetic association between exposure and outcome was explained by linkage disequilibrium [39–41]. The colocalization analysis yielded several posterior probability hypotheses (PPH) [41]:(1)No genetic association between the two traits (PPH0); (2) Only trait 1 had a genetic association (PPH1); (3) Only trait 2 had a genetic association (PPH2); (4) The two traits were correlated but had different causal variables (PPH3); (5) The two traits shared a causal variant, with the first trait influencing the second trait (PPH4). In this study, we conducted colocalization analysis using all SNP data within the entire cis-coding region of pQTL (± 500Kb). To satisfy the causal hypothesis, PPH4 commonly required a threshold of 80% [42].

### Replication in deCODE

The cis-pQTL for PCSK9 was derived from the deCODE study, and the IVs were chosen based on the more stringent selection criteria previously outlined. For two-sample MR, the principal method employed was the IVW approach, supplemented by the weighted median and MR-Egger techniques. Additional sensitivity analyses were performed to evaluate heterogeneity using Cochran’s Q test, and to identify potential horizontal pleiotropy through Egger regression and MR-PRESSO. Subsequently, the colocalization method was employed to enhance the reliability of the causality.

### Multivariable MR and two-step MR

Multivariable MR was performed to investigate the potential mediating role of common risk factors associated with HF, including coronary heart disease (CHD), body mass index (BMI), high-density lipoprotein cholesterol (HDL-C), low-density lipoprotein cholesterol (LDL-C), systolic blood pressure (SBP), and diastolic blood pressure (DBP), in the observed relationship. Each risk factor was individually examined for its mediating effect [43]. Genetic instruments for each trait were obtained and harmonized with those for circulating PCSK9 (Tables S5). The IVW method was utilized to account for potential mediators in the multivariable MR model. To evaluate the potential mediating influence of CHD or LDL-C, an additional mediation strategy, known as the two-step MR approach, was employed. This approach utilized the coefficients method to estimate the indirect effect (β1 × β2) and the proportion of mediation (Indirect effect / Total effect) attributed to mediators [44].

### Analysis of the single-cell sequencing results

We conducted an analysis of cardiac cells PCSK9 expression levels using single-cell RNA-seq data obtained from Bridget Simonson et al. [45]. The raw data can be accessed and visualized on the Single Cell Portal (https://singlecell.broadinstitute.org/single_cell). The dataset consisted of 99,684 cells derived from 7 recipients with ischemic cardiomyopathy (ICM) at end-stage HF and 8 non-failing (NF) controls. By examining the expression of PCSK9, we determined the PCSK9 expression level in each cell within the myocardial tissue and evaluated the expression differences between the disease and control groups.

### Software

The study was conducted using R 4.2.2 software, with a primary focus on utilizing the following R packages: “MendelianRandomization” (version 0.7.0), “TwoSampleMR” (version 0.5.6), “MRPRESSO” (version 1.0), “coloc” (version 5.1.0.1), and “MVMR” (version 0.3). To establish statistical significance, a threshold of *P* < 0.05 was employed.

## Results

### Causal effect estimation of circulating proteins on heart failure

**Fig. 2:**
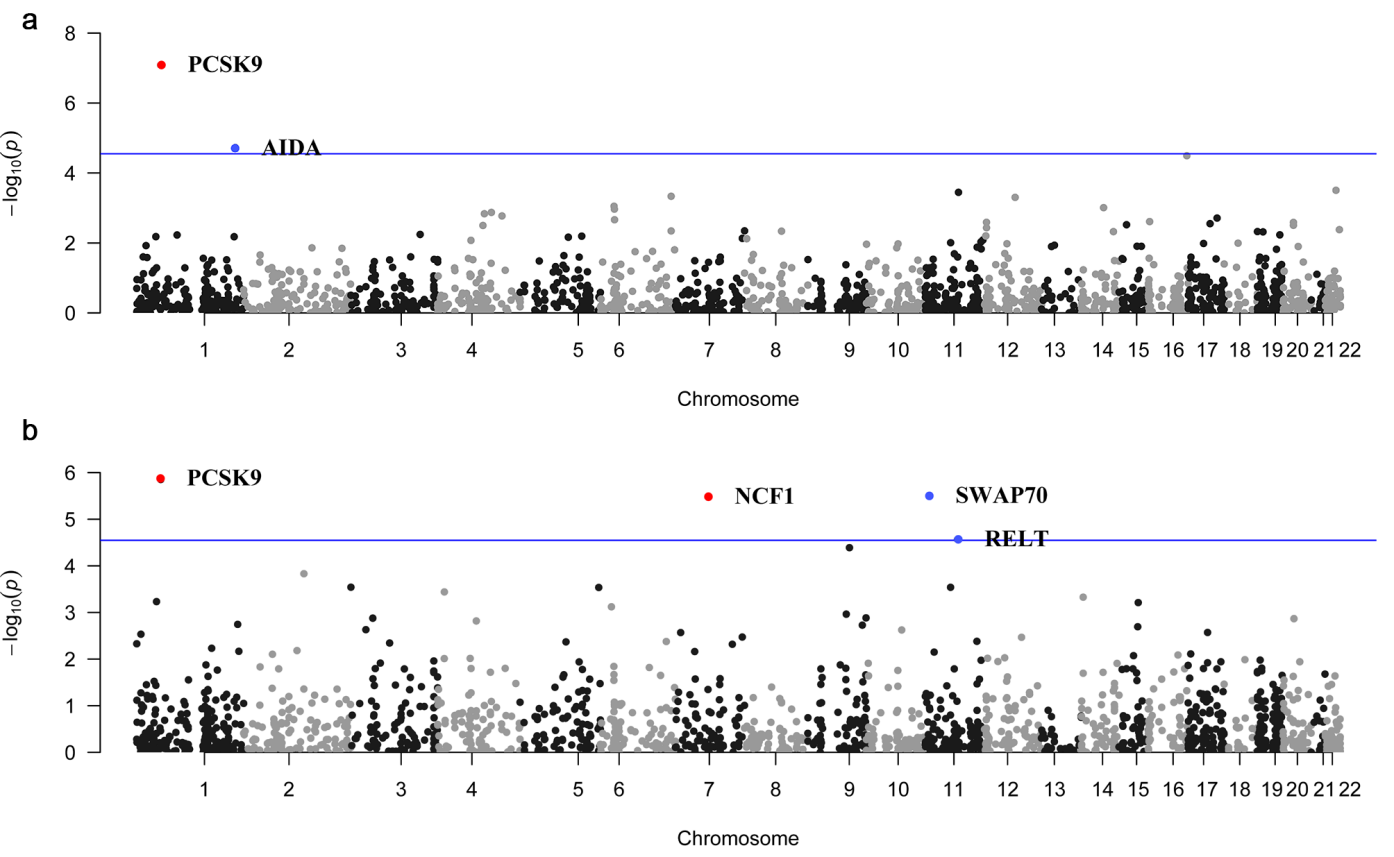
Manhattan plots for associations of genetically predicted circulating proteins levels with HF in MR analysis. (**a**) Associations of genetically predicted circulating proteins with HF in patients with CHD; (**b**) Associations of genetically predicted circulating proteins with HF in patients receiving antihypertensive medication. Labelled and colored proteins refer to MR findings with Bonferroni-corrected *P* < 0.05 (two-sample MR analysis). Red proteins indicate the positive effect of the circulating proteins on outcomes; blue proteins indicate the negative effect of the circulating proteins on outcomes; blue line indicates the Bonferroni-corrected *P* value equals to 0.05. Results are plotted by gene start position

We performed proteome-wide Mendelian Randomization (MR) analyses to examine the associations between proteins with available index pQTL signals and the risk of HF outcomes. The IVW results showed no genetically predicted higher levels of circulating proteins associated with all-cause HF, regardless of the source of outcome data. (Figure S1, Table S6-7). However, we observed significant associations between genetically predicted higher levels of 2 circulating proteins and HF in patients with CHD (namely PCSK9 and AIDA) (Fig. 2, Table S8). Specifically, genetically predicted PCSK9, with three SNPs as IVs, exhibited a positive association with the risk of HF in patients with CHD (OR: 1.273, 95% CI: 1.165–1.390, BF-corrected *p*-value: 1.45E-04). Conversely, genetically predicted AIDA, with only one SNP as the genetic instrument, showed a negative association with the risk of HF in patients with CHD (Table 1, Table S8). Additionally, our findings revealed genetically predicted higher levels of 4 circulating proteins related with HF in patients receiving antihypertensive medication (namely PCSK9, SWAP70, NCF1, and RELT) (Fig. 2, Table S9). Among these findings, PCSK9 was positive with the risk of HF in patients receiving antihypertensive medication (OR: 1.145, 95% CI: 1.084–1.210, BF-corrected *p*-value: 2.50E-03) (Table 1, Table S9). The Cochran’s Q test and Egger regression intercept tests showed no evidence of heterogeneity or horizontal pleiotropy for PCSK9 and others (Table S10). Furthermore, supplementary methods yielded consistent directional effects for these proteins (Table S10). However, there were insufficient SNPs available for AIDA and RELT to perform sensitivity analyses and supplementary methods.

To confirm these associations, we conducted colocalization analysis. The results consistently supported the correlation between PCSK9 and HF in patients with CHD (PPH4: 99.998%), as well as between PCSK9 and HF in patients receiving antihypertensive medication (PPH4: 98.864%) (Table 1) Additionally, we observed a robust colocalization evidence for SWAP70 and HF in patients receiving antihypertensive medication (81.718%). However, NCF1 did not meet the PPH4 criteria, which was below 80% (PPH4: 60.047%) (Table 1). Complete colocalization results were provided in Table S11.

**Table 1:** Mendelian randomization and colocalization analyses of circulating proteins on HF outcomes: Insights from the ARIC dataset. HF: heart failure; CHD: Coronary heart disease; CI, Confidence interval; BF, Bonferroni; OR, Odds ratio

Outcomes	Proteins	OR (95% CI)	*P* value	BF-corrected *P* value	Colocalization Analysis P_H4_ (%)
HF in patients with CHD	PCSK9	1.273 (1.165–1.390)	8.18E-08	1.45E-04	99.998
HF in patients with CHD	AIDA	0.768 (0.681–0.867)	1.88E-05	3.33E-02	92.621
HF in patients receiving antihypertensive medication	PCSK9	1.145 (1.084–1.210)	1.41E-06	2.50E-03	98.864
HF in patients receiving antihypertensive medication	SWAP70	0.906 (0.870–0.945)	3.10E-06	5.50E-03	81.718
HF in patients receiving antihypertensive medication	NCF1	1.100 (1.057–1.145)	3.23E-06	5.74E-03	60.047
HF in patients receiving antihypertensive medication	RELT	0.697 (0.589–0.825)	2.72E-05	4.82E-02	93.461

### Causal effect estimation of circulating proteins on ischemic heart disease and essential hypertension

Given the presence of case overlap between HF in patients with CHD and HF in patients receiving antihypertensive medication (47.70%) (Table S3), it is difficult to tell whether circulating PCSK9 is associated with ischemic HF or hypertensive HF. Consequently, we proceeded with supplementary proteome-wide MR analyses to investigate its association with different HF etiologies. The study investigated the Mendelian Randomization (MR) associations between proteins possessing accessible index pQTL signals and the risk of the common etiologies of HF, including ischemic heart disease (IHD) and essential hypertension. Genetically predicted higher levels of 10 circulating proteins were significantly associated with a decreased risk of IHD (Figure S2). Among these proteins, genetically predicted PCSK9 was the most significant related to IHD (OR: 1.231, 95% CI: 1.162–1.304, BF-corrected *p*-value: 2.53E-09). Additionally, genetically predicted higher levels of 16 circulating proteins were significantly associated with a decreased risk of essential hypertension (Figure S3). Complete proteome-wide MR results were provided in Table S12-13.

### Replication in deCODE

To further validate the discovered relationships, we conducted replication analyses using pQTL data from the deCODE study. The IVW result showed significant associations between genetically predicted PCSK9 and HF in patients with CHD (OR: 1.320, 95% CI: 1.226–1.421, *p*-value: 1.56E-13) (Table 2). The Cochran’s Q test and Egger regression intercept tests showed no evidence of heterogeneity or horizontal pleiotropy. Moreover, both the weighted median and MR-Egger methods provided effect estimates that aligned with the IVW result (Table 2). Additionally, the colocalization analysis provided compelling evidence supporting the correlation between genetically predicted PCSK9 and HF in patients with CHD (99.993%) (Table 2). Complete colocalization results can be found in Table S11. In contrast to PCSK9, the relationship between genetically predicted SWAP70 and HF in patients receiving antihypertensive medication was not validated in replication (Table S14).

**Table 2:** Mendelian randomization and colocalization analyses of circulating PCSK9 on HF in patients with CHD: Insights from the deCODE. dataset. HF: heart failure; CHD: Coronary heart disease; CI, Confidence interval; OR, Odds ratio

Outcome	Methods	OR (95% CI)	*P* value	Colocalization Analysis P_H4_ (%)
HF in patients with CHD	IVW (P for heterogeneity = 0.560)	1.320 (1.226–1.421)	1.56E-13	99.993
	Weighted median	1.317 (1.213–1.429)	4.72E-11	
	MR-Egger	1.273 (1.141–1.420)	0.023	
	MR-Egger regression		0.445	
	MR-PRESSO (no outliers detected)		0.613	

### Evaluating the mediating effects of HF-related risk factors

**Fig. 3:**
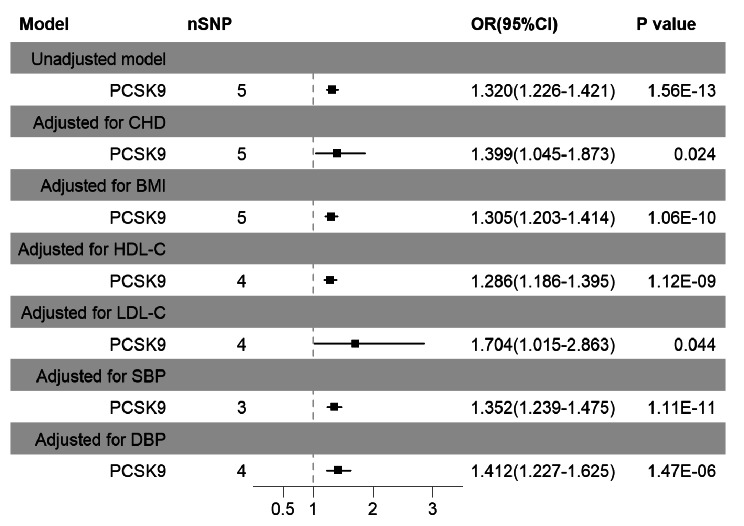
Multivariable MR analysis for PCSK9 and HF in patients with CHD after adjusting HF-related risk factor. SNP: Single nucleotide polymorphism; HF: heart failure; CHD: Coronary heart disease; BMI: Body mass index; HDL-C: High density lipoprotein cholesterol; LDL-C: Low density lipoprotein cholesterol; SBP: Systolic blood pressure; DBP: Diastolic blood pressure; CI, Confidence interval; OR, Odds ratio

To evaluate the potential mediating role of common risk factors associated with HF, including CHD, BMI, HDL-C, LDL-C, SBP, and DBP, we conducted multivariable MR analyses. Importantly, after MVMR adjustment for CHD or LDL-C, the significant association between PCSK9 and HF in patients with CHD persisted, suggesting that CHD and LDL-C may not exert a complete mediating effect in this relationship. However, it is crucial to note that after adjusting for LDL-C, the association between genetically predicted PCSK9 and HF in patients with CHD approached null (*P* = 0.044), warranting cautious interpretation of this result. Adjustments for other risk factors such as BMI, HDL-C, SBP, or DBP yielded similar associations (Fig. 3).

To assess the proportion of the mediating effect attributable to CHD or LDL-C, we conducted two-step MR analyses. Given the potential pleiotropy in CHD genetic instruments, we employed the PRESSO method to identify and exclude two SNPs with significant impacts, enhancing the internal consistency and reliability of the MR study (Table S5). In the PCSK9-CHD-outcome mediation pathway, the indirect effect was estimated at 0.179, with LDL-C accounting for 64.494% of the total mediated effect (Figure S4). Additionally, we acquired genetic instruments for LDL-C, excluding three SNPs that overlapped with those of PCSK9 (Table S5). In the PCSK9-LDL-C-outcome mediation pathway, the indirect effect was calculated as 0.147, and the proportion mediated by LDL-C was 52.792% (Figure S5). Full details of the two-step MR analyses are available in Table S15-16.

### Analyzing PCSK9 expression levels in cardiac cells

To assess the expression of PCSK9 in cardiac cells within the context of ischemic heart failure (HF), we analyzed PCSK9 protein expression in end-stage ischemic cardiomyopathy (ICM) and compared it to non-failing (NF) controls using the Single Cell Portal. The findings revealed no noteworthy disparities in PCSK9 expression between the two groups, and PCSK9 exhibited limited expression across various cell types (Figure S6-7).

## Discussion

This study aimed to investigate the relationships between genetically predicted elevated levels of circulating proteins and various heart failure (HF) outcomes, as well as their respective etiologies, using a proteome-wide Mendelian randomization (MR) approach. Our study identified two proteins, PCSK9 and AIDA, linked to HF in patients with coronary heart disease (CHD), and four proteins, PCSK9, SWAP70, NCF1, and RELT, associated with HF in patients receiving antihypertensive medication. Comprehensive sensitivity and colocalization analyses were conducted for all results. Furthermore, supplementary proteome-wide MR analyses were executed to scrutinize the association of genetically predicted proteins with specific HF etiologies, with the aim of validating potential overlaps between proteins associated with HF outcomes and their respective etiologies. These analyses collectively provided robust evidence indicating that genetically predicted higher levels of circulating PCSK9 was associated with an increased risk of ischemic heart disease (IHD) and HF in patients with CHD. Furthermore, our results demonstrated that predicted higher levels of circulating SWAP70 were associated with an increased risk of essential hypertension and HF in patients receiving antihypertensive medication. Subsequently, we validated these findings using independent pQTL data from the deCODE study, employing a two-sample MR approach and conducting sensitivity analyses during the replication phase. Moreover, our multivariable MR and two-step MR analyses were conducted to reveal potential partial mediators.

Recently, several studies have focused on identifying therapeutic targets for HF through the proteome-wide MR approach [46–48]. However, these studies have primarily focused on all-cause HF. And there were no duplicate proteins in the results of these studies (Table S16). In a recent plasma proteome Genome-Wide Association Study (GWAS) conducted in a population of European ancestry from the Atherosclerosis Risk in Communities (ARIC) study, independent protein quantitative trait loci (pQTLs) for 4,657 proteins were identified in 7,213 individuals [30]. To enhance the reliability of potential target proteins identified in previously published studies, we conducted proteome-wide MR analysis using this latest proteome data in the context of all-cause HF. Surprisingly, our results did not yield any significant proteins, both within the HERMES Consortium and the FinnGen Consortium. The discrepancy between our results and previously published findings may be attributed to following factors. Firstly, the proteome-wide MR analyses in the published studies obtained pQTL data from various sources, encompassing 40, 2,965, and 2,994 proteins, respectively (Table S16). Although our study included a larger number of proteins, there may still be deficiencies in certain proteins. Secondly, the selection criteria for genetic instruments in the proteome-wide MR analyses of the published studies were more lenient compared to the criteria utilized in our study (Table S16).

HF is a complex clinical syndrome with various underlying causes, primarily categorized as either ischemic or non-ischemic [49]. Tailoring treatment strategies based on HF etiology is crucial [49]. However, there is a paucity of MR studies addressing this aspect. In our study, we made extensive efforts to gather publicly available GWAS data. Ultimately, we selected two large GWAS datasets from the FinnGen Consortium: HF in patients with CHD and HF in patients receiving antihypertensive medication, respectively. Utilizing proteome-wide Mendelian randomization (MR) analyses, we identified several proteins associated with these outcomes. In alignment with our hypothesis concerning PCSK9, we observed a positive association with HF in patients with CHD. Surprisingly, we also identified a PCSK9-outcome link for HF in patients under antihypertensive medication. In addition to PCSK9, our study uncovered several potential targets. Specifically, AIDA displayed a negative correlation with HF in patients with CHD. Moreover, we observed that SWAP70 and RELT were negatively associated with an elevated risk of HF in patient receiving antihypertensive medication, while NCF1 demonstrated a positive correlation. These findings expand our understanding of potential therapeutic targets for HF. Nonetheless, cautious interpretation of these findings is essential. Firstly, as supplementary methods, the weighted median and MR-Egger results generally aligned with the IVW results for most protein-outcome causality associations. However, for AIDA and RELT, only one SNP remained as the genetic instrument after selection, limiting supplemental validation. Secondly, evaluating potential pleiotropy influencing protein-disease causality associations was essential. The Egger regression analysis indicated that the majority of causality results were unlikely affected by horizontal pleiotropy. However, due to limited availability of genetic instruments, the impact of horizontal pleiotropy on the results for AIDA and RELT remained uncertain. Thirdly, the colocalization analysis revealed that the association between NCF1 and HF in patients receiving antihypertensive medication might be influenced by linkage disequilibrium rather than a direct causal link [39–41]. Overall, during the discovery stage, the primary findings consistently indicate that PCSK9 holds promise as an intervention target in HF. Additionally, it was observed that genetically predicted elevated levels of circulating SWAP70 were associated with a decreased risk of essential hypertension and HF in patients receiving antihypertensive medication. During the replication stage, we validated the association between genetically predicted elevated levels of circulating PCSK9 and HF in patients with CHD. However, the association between genetically predicted elevated levels of circulating SWAP70 and HF in patients receiving antihypertensive medication did not align with the results from the discovery stage (Table S14). Consequently, our subsequent analyses and discussions primarily focused on PCSK9.

Circulating PCSK9 has emerged as a potential therapeutic target for HF [50, 51]. However, recent studies have presented conflicting findings [15, 17–19]. Although previous two-sample MR studies based on GWAS of lipid traits or eQTL of PCSK9 have provided support for the association between PCSK9 and all-cause HF [24–26], our study found no evidence of the correlation between circulating PCSK9 and the risk of all-cause HF. This aligns with previously published proteome-wide MR studies [46–48]. Consistent with our findings, a clinical observational study that adjusted for confounding factors, including age, sex, and diabetes, also reported no association between PCSK9 and all-cause HF [15]. Additionally, meta-analyses and a recent randomized controlled trial (RCT) study, which provide higher-level clinical evidence, consistently concluded that the use of PCSK9 inhibitors did not significantly affect all-cause HF [17–19]. Notably, another MR study failed to establish a relationship between PCSK9 and HF in patients with non-ischemic cardiovascular disease (CVD) events, leaving the association between PCSK9 and ischemic HF uncertain [27]. To comprehensively investigate potential effects of PCSK9 in relation to ischemic HF, we extended our investigation by conducting additional proteome-wide MR analyses across diverse HF etiologies (ischemic heart disease and essential hypertension). CHD is a specific subtype of ischemic heart disease (IHD) characterized by the presence of atherosclerosis in the coronary arteries, and angina pectoris or myocardial infarction (MI) are considered as common clinical symptoms [52]. Our findings demonstrated a positive association between genetically predicted elevated levels of circulating PCSK9 and the risk of IHD, implying a potential protective effect of PCSK9 inhibitors in the context of ischemic HF. In contrast, no significant association was observed between genetically predicted PCSK9 and essential hypertension. This suggests that the apparent link between PCSK9 and HF in patients receiving antihypertensive medication might be influenced by the inclusion of patients with CHD, rather than indicating a genuine relationship with hypertensive HF. Consistently, a prior descriptive cohort study revealed that PCSK9 inhibitors were predominantly used in patients with IHD in clinical practice [53]. Likewise, a recent observational study identified a correlation between circulating PCSK9 levels and left ventricular ejection fraction (LVEF) after MI [12, 13]. Furthermore, another observational study, which included a significant proportion of patients with IHD, established a link between PCSK9 and the prognosis of HF [14]. Taken together, our proteome-wide MR results and these previous findings indicate the involvement of PCSK9 in the development of ischemic HF.

In the discovery stage, we have provided evidence supporting the association between PCSK9 and the development of HF in patients with CHD. To validate this finding, we conducted replication analyses using an independent pQTL data from the deCODE study. This dataset consisted of pQTLs for 4,907 blood proteins measured in 35,559 individuals of European ancestry from Iceland [31]. With an ample number of instrumental variables, we performed cis-MR analysis and conducted sensitivity analyses. The replication results were consistent with those of discovery stage, reinforcing the evidence for a relationship between circulating PCSK9 and an elevated risk of HF in patients with CHD.

While our study consistently indicates an association between genetically predicted PCSK9 and HF in patients with CHD, considering the potential linkage between PCSK9 and CHD, it is insufficient to disentangle the effects of PCSK9 on HF and CHD. Consequently, we supplemented our analysis with MVMR analyses to explore the potential mediating effect of CHD on the association between circulating PCSK9 and HF in patients with CHD. Our investigation reveals that even after adjusting for CHD, the connection between PCSK9 and concomitant HF and CHD remains significant, suggesting that CHD may not play a complete mediating role in the association. Further two-step MR analysis identified the mediating effect proportion of CHD to be 64.494%. Similar results were found in the LDL-C mediation analysis. In summary, our study results imply that PCSK9 may influence the progression of ischemic HF through pathways beyond LDL-C/CHD.

Beyond its lipid-modulating role, researchers have been intrigued by how PCSK9 impacts the progression of ischemic HF. In 2017, Klaus-Dieter Schlüter’s team first reported the expression of PCSK9 in cardiomyocyte [54]. In 2018, Zufeng Ding’s team highlighted elevated expression of PCSK9 in hypoxic cardiomyocyte and the border zone of a mouse myocardial infarction model, with an induction of autophagy dependent on extracellular PCSK9 concentrations [55]. Subsequently, the roles and mechanisms of PCSK9 in autophagy, apoptosis, and necroptosis in cardiomyocyte have been progressively discovered [56–61]. It is noteworthy that current research predominantly focuses on cardiomyocyte damage during the ischemia-reperfusion injury or acute myocardial infarction, i.e., the ischemic necrotic phase. Limited reports are available regarding the relationship between PCSK9 and the subsequent stages of ventricular remodeling (fibrotic and scar formation periods). In 2023, Chen Wu and colleagues reported for the first time that a PCSK9 inhibitor can ameliorate post-infarction ventricular remodeling. The study demonstrated, through the Notch1 signaling pathway, the inhibitory effect of PCSK9 derived from cardiac fibroblasts on hypoxia-induced cardiac fibroblast phenotypic transformation [62]. Collectively, these studies propose that, beyond its lipid-modulating role, PCSK9 may influence HF progression by participating in myocardial cell death patterns and abnormal activation of cardiac fibroblasts, leading to ventricular remodeling and, consequently, direct involvement in HF progression.

Furthermore, it is of significant importance to examine the distinction between extracellular and intracellular endogenous PCSK9 within the context of HF. Upon conducting an online analysis of the single-cell sequencing results, we identified restricted PCSK9 expression within human cardiac cells, which was observed in both ischemic cardiomyopathy (ICM) at end-stage HF and non-failing (NF) controls. This finding suggests a potential divergence in the biological roles of extracellular and intracellular endogenous PCSK9. Recent research has revealed that cardiomyocyte-derived PCSK9 exerts a suppressive effect on both cardiac systolic and diastolic functions through an autocrine mechanism. This inhibition can be mitigated by the PCSK9 inhibitor Alirocumab [63]. Conversely, another study found that the cardiac-specific knockout of PCSK9 led to the development of heart failure with preserved ejection fraction (HFpEF) [64]. Collectively, these findings suggest that focusing on extracellular PCSK9 inhibition, rather than intracellular PCSK9 inhibition, might serve as a viable intervention strategy for ischemic HF.

Nowadays, Alirocumab and Evolocumab are widely utilized PCSK9 inhibitors in clinical settings [65, 66], and several new inhibitors are currently under development (Table S17). These inhibitors provide a convenient approach to investigating the therapeutic mechanism of PCSK9 in ischemic HF. However, among these inhibitors, only Evolocumab has advanced to phase II clinical trials for ischemic HF (Table S17). Interestingly, a recent study based on the EVO-HF trial revealed that Evolocumab potentially leading to upregulation of the PCSK9 gene expression [67]. Ethical considerations in clinical studies preclude the exclusive use of PCSK9 inhibitors in ischemic HF patients for investigating circulating PCSK9 levels, as well as for conducting diagnostic and prognostic studies. Therefore, further animal experiments involving hepatic PCSK9 knockout are necessary to clarify the inhibitory effect of PCSK9 on ischemic HF.

The strength of our study lies in the utilization of proteome-wide MR and colocalization analyses to investigate the potential causal relationship between circulating proteins and various HF outcomes. Furthermore, the finding was validated through replication analyses. However, it is essential to acknowledge several limitations. Firstly, in the discovery stage, we only identified six significant proteins. This may be attributed to the implementation of more stringent selection criteria for genetic instruments compared to published proteome-wide MR studies, potentially resulting in the non-detection of certain proteins. Secondly, while our findings supported a potential causal relationship between PCSK9 and HF in patients with CHD, suggesting its potential as a therapeutic target for ischemic HF, it is important to recognize the complex of HF. Thus, our study falls short of definitively establishing the causal role of PCSK9 in patients with complex complications and medications. Thirdly, it is crucial to note that the MR analysis exclusively included participants of European descent, limiting the generalizability of our findings to other populations. Caution should be exercised when extrapolating these results to different ethnic or geographic groups. Fourthly, despite our mediation analyses and recent basic research suggesting that PCSK9 may impact the progression of ischemic HF through pathways beyond LDL-C/CHD, achieving a conclusive understanding requires employing individual GWAS datasets or conducting clinical trials in specific patient cohorts. Lastly, the pQTL data used in our study are all from the SomaScan platform (ARIC study and deCODE study). Recent research suggests that pQTL data obtained from the Olink platform may have advantages in terms of protein target specificity, phenotypic associations, and accuracy in repeated measurements [68, 69]. Therefore, incorporating data from different platforms helps researchers better understand the relationship between proteins and diseases and may identify new potential related proteins.

## Conclusions

We conducted a comprehensive investigation into the potential causal relationship between PCSK9 and various HF outcomes. The findings demonstrated a significant association between PCSK9 and concomitant HF and CHD. Furthermore, our mediation analyses suggest that PCSK9 may impact the progression of ischemic HF through pathways beyond LDL-C/CHD. These outcomes offer valuable insights to propel clinical trials involving PCSK9 inhibitors in the context of ischemic HF.

### Electronic supplementary material

Below is the link to the electronic supplementary material.

Supplementary Material 1

Supplementary Material 2

## Data Availability

The pQTL GWAS summary statistics were derived from the ARIC study and the deCODE study, while the other GWAS summary statistics data were sourced from publicly available websites. It is important to note that all the supporting data used in our MR analyses do not include any personal identifying information of participants and are accessible through the web sources listed in Table S2. For the complete code used in our study, interested individuals can request it from the corresponding author.

## Abbreviations

HF: Heart failure
MR: Mendelian randomization
CHD: Coronary heart disease
pQTL: Protein quantitative trait loci
ARIC: Atherosclerosis Risk in Communities
deCODE, Diabetes epidemiology: Collaborative analysis of diagnostic criteria in Europe
PCSK9: Protein convertase subtilisin/kexin type 9
LDLR: Low-density lipoprotein receptor
LVEF: Left ventricular ejection fraction
MI: Myocardial infarction
RCTs: Randomized controlled trials
MACE: Major adverse cardiovascular events
HFrEF: Heart failure with reduced ejection fraction
IVs: Instrumental variables
CVD: Cardiovascular disease
GWAS: Genome-Wide Association Study
eQTL: Expression quantitative trait loci
STROBE-MR: Strengthening the Reporting of Observational Studies in Epidemiology using Mendelian Randomization
PCs: Principal components
TSS: Transcription start site
ICD: International Classification of Disease
IHD: Ischemic heart disease
IVs: Instrumental variables
LD: Linkage disequilibrium
SNP: Single nucleotide polymorphism
IVW: Inverse variance weighted
BF: Bonferroni
PPH: Posterior probability hypotheses
BMI: Body mass index
HDL-C: High-density lipoprotein cholesterol
LDL-C: Low-density lipoprotein cholesterol
SBP: Systolic blood pressure
DBP: Diastolic blood pressure
ICM: Ischemic cardiomyopathy
NF: Non-failing
OR: Odds ratio
CI: Confidence interval
HFpEF: Heart failure with preserved ejection fraction

## References

[1] Metra M, Teerlink JR (2017). Heart failure. Lancet.

[2] McDonagh TA, Metra M, Adamo M, Gardner RS, Baumbach A, Böhm M (2022). 2021 ESC guidelines for the diagnosis and treatment of acute and chronic heart failure: developed by the Task Force for the diagnosis and treatment of acute and chronic heart failure of the European Society of Cardiology (ESC) with the special contribution of the Heart Failure Association (HFA) of the ESC. Rev Esp Cardiol (Engl Ed).

[3] Roger VL, Weston SA, Redfield MM, Hellermann-Homan JP, Killian J, Yawn BP (2004). Trends in heart failure incidence and survival in a community-based population. JAMA.

[4] Glynn PA, Ning H, Bavishi A, Freaney PM, Shah S, Yancy CW (2021). Heart failure risk distribution and Trends in the United States Population, NHANES 1999–2016. Am J Med.

[5] Ge Y, Wang TJ (2012). Identifying novel biomarkers for cardiovascular disease risk prediction. J Intern Med.

[6] Cao TH, Jones DJL, Voors AA, Quinn PA, Sandhu JK, Chan DCS (2020). Plasma proteomic approach in patients with heart failure: insights into pathogenesis of disease progression and potential novel treatment targets. Eur J Heart Fail.

[7] Shimada YJ, Cannon CP (2015). PCSK9 (Proprotein convertase subtilisin/kexin type 9) inhibitors: past, present, and the future. Eur Heart J.

[8] Benhuri B, Ueyama H, Takagi H, Briasoulis A, Kuno T (2021). PCSK9 inhibitors and Ezetimibe Monotherapy in patients not receiving statins: a Meta-analysis of Randomized trials. Curr Vasc Pharmacol.

[9] Hao Q, Aertgeerts B, Guyatt G, Bekkering GE, Vandvik PO, Khan SU (2022). PCSK9 inhibitors and ezetimibe for the reduction of cardiovascular events: a clinical practice guideline with risk-stratified recommendations. BMJ.

[10] Schwartz GG, Steg PG, Szarek M, Bhatt DL, Bittner VA, Diaz R (2018). Alirocumab and Cardiovascular outcomes after Acute Coronary Syndrome. N Engl J Med.

[11] Sabatine MS, Giugliano RP, Keech AC, Honarpour N, Wiviott SD, Murphy SA (2017). Evolocumab and Clinical outcomes in patients with Cardiovascular Disease. N Engl J Med.

[12] Bayes-Genis A, Núñez J, Zannad F, Ferreira JP, Anker SD, Cleland JG (2017). The PCSK9-LDL receptor Axis and outcomes in Heart failure: BIOSTAT-CHF subanalysis. J Am Coll Cardiol.

[13] Miñana G, Núñez J, Bayés-Genís A, Revuelta-López E, Ríos-Navarro C, Núñez E (2020). Role of PCSK9 in the course of ejection fraction change after ST-segment elevation myocardial infarction: a pilot study. ESC Heart Fail.

[14] Silva-Bermúdez LS, Vargas-Villanueva A, Sánchez-Vallejo CA, Palacio AC, Buitrago AF, Mendivil CO (2022). Peri-event plasma PCSK9 and hsCRP after an acute myocardial infarction correlate with early deterioration of left ventricular ejection fraction: a cohort study. Lipids Health Dis.

[15] Bouwens E, Schuurman AS, Akkerhuis KM, Manintveld OC, Caliskan K, van Ramshorst J (2021). Associations of serially measured PCSK9, LDLR and MPO with clinical outcomes in heart failure. Biomark Med.

[16] Niessner A, Drexel H (2022). PCSK9 inhibition in patients with heart failure: neutral or harmful intervention?. Eur Heart J.

[17] Du H, Li X, Su N, Li L, Hao X, Gao H (2019). Proprotein convertase subtilisin/kexin 9 inhibitors in reducing cardiovascular outcomes: a systematic review and meta-analysis. Heart.

[18] van Bruggen FH, Nijhuis GBJ, Zuidema SU, Luijendijk H (2020). Serious adverse events and deaths in PCSK9 inhibitor trials reported on ClinicalTrials.gov: a systematic review. Expert Rev Clin Pharmacol.

[19] White HD, Schwartz GG, Szarek M, Bhatt DL, Bittner VA, Chiang CE (2022). Alirocumab after acute coronary syndrome in patients with a history of heart failure. Eur Heart J.

[20] Sekula P, Del Greco MF, Pattaro C, Köttgen A (2016). Mendelian randomization as an Approach to assess causality using Observational Data. J Am Soc Nephrol.

[21] Grover S, Del Greco MF, Stein CM, Ziegler A (2017). Mendelian randomization. Methods Mol Biol.

[22] Bowden J, Holmes MV (2019). Meta-analysis and mendelian randomization: a review. Res Synth Methods.

[23] Davey Smith G, Hemani G (2014). Mendelian randomization: genetic anchors for causal inference in epidemiological studies. Hum Mol Genet.

[24] Schmidt AF, Hunt NB, Gordillo-Marañón M, Charoen P, Drenos F, Kivimaki M (2021). Cholesteryl Ester transfer protein (CETP) as a drug target for cardiovascular disease. Nat Commun.

[25] Cupido AJ, Reeskamp LF, Hingorani AD, Finan C, Asselbergs FW, Hovingh GK (2022). Joint genetic inhibition of PCSK9 and CETP and the Association with Coronary Artery Disease: a factorial mendelian randomization study. JAMA Cardiol.

[26] Xiao J, Ji J, Zhang N, Yang X, Chen K, Chen L (2023). Association of genetically predicted lipid traits and lipid-modifying targets with heart failure. Eur J Prev Cardiol.

[27] Schmidt AF, Holmes MV, Preiss D, Swerdlow DI, Denaxas S, Fatemifar G (2019). Phenome-wide association analysis of LDL-cholesterol lowering genetic variants in PCSK9. BMC Cardiovasc Disord.

[28] Schmidt AF, Finan C, Gordillo-Marañón M, Asselbergs FW, Freitag DF, Patel RS (2020). Genetic drug target validation using mendelian randomisation. Nat Commun.

[29] Skrivankova VW, Richmond RC, Woolf BAR, Davies NM, Swanson SA, VanderWeele TJ (2021). Strengthening the reporting of observational studies in epidemiology using mendelian randomisation (STROBE-MR): explanation and elaboration. BMJ.

[30] Zhang J, Dutta D, Köttgen A, Tin A, Schlosser P, Grams ME (2022). Plasma proteome analyses in individuals of European and African ancestry identify cis-pQTLs and models for proteome-wide association studies. Nat Genet.

[31] Ferkingstad E, Sulem P, Atlason BA, Sveinbjornsson G, Magnusson MI, Styrmisdottir EL (2021). Large-scale integration of the plasma proteome with genetics and disease. Nat Genet.

[32] Zhao H, Rasheed H, Nøst TH, Cho Y, Liu Y, Bhatta L (2022). Proteome-wide mendelian randomization in global biobank meta-analysis reveals multi-ancestry drug targets for common diseases. Cell Genom.

[33] Burgess S, Thompson SG (2011). Avoiding bias from weak instruments in mendelian randomization studies. Int J Epidemiol.

[34] Burgess S, Small DS, Thompson SG (2017). A review of instrumental variable estimators for mendelian randomization. Stat Methods Med Res.

[35] Burgess S, Butterworth A, Thompson SG (2013). Mendelian randomization analysis with multiple genetic variants using summarized data. Genet Epidemiol.

[36] Burgess S, Bowden J, Fall T, Ingelsson E, Thompson SG (2017). Sensitivity analyses for robust causal inference from mendelian randomization analyses with multiple genetic variants. Epidemiology.

[37] Kulinskaya E, Dollinger MB (2015). An accurate test for homogeneity of odds ratios based on Cochran’s Q-statistic. BMC Med Res Methodol.

[38] Bowden J, Del Greco MF, Minelli C, Davey Smith G, Sheehan NA, Thompson JR (2016). Assessing the suitability of summary data for two-sample mendelian randomization analyses using MR-Egger regression: the role of the I2 statistic. Int J Epidemiol.

[39] Giambartolomei C, Vukcevic D, Schadt EE, Franke L, Hingorani AD, Wallace C (2014). Bayesian test for colocalisation between pairs of genetic association studies using summary statistics. PLoS Genet.

[40] Giambartolomei C, Zhenli Liu J, Zhang W, Hauberg M, Shi H, Boocock J (2018). A bayesian framework for multiple trait colocalization from summary association statistics. Bioinformatics.

[41] Foley CN, Staley JR, Breen PG, Sun BB, Kirk PDW, Burgess S (2021). A fast and efficient colocalization algorithm for identifying shared genetic risk factors across multiple traits. Nat Commun.

[42] Zuber V, Grinberg NF, Gill D, Manipur I, Slob EAW, Patel A (2022). Combining evidence from mendelian randomization and colocalization: review and comparison of approaches. Am J Hum Genet.

[43] Burgess S, Thompson SG (2015). Multivariable mendelian randomization: the use of pleiotropic genetic variants to estimate causal effects. Am J Epidemiol.

[44] Carter AR, Sanderson E, Hammerton G, Richmond RC, Davey Smith G, Heron J (2021). Mendelian randomisation for mediation analysis: current methods and challenges for implementation. Eur J Epidemiol.

[45] Simonson B, Chaffin M, Hill MC, Atwa O, Guedira Y, Bhasin H (2023). Single-nucleus RNA sequencing in ischemic cardiomyopathy reveals common transcriptional profile underlying end-stage heart failure. Cell Rep.

[46] Henry A, Gordillo-Marañón M, Finan C, Schmidt AF, Ferreira JP, Karra R (2022). Therapeutic targets for heart failure identified using proteomics and mendelian randomization. Circulation.

[47] Moncla LM, Mathieu S, Sylla MS, Bossé Y, Thériault S, Arsenault BJ (2022). Mendelian randomization of circulating proteome identifies actionable targets in heart failure. BMC Genomics.

[48] Yang J, Yan B, Zhang H, Lu Q, Yang L, Liu P (2023). Estimating the causal effects of genetically predicted plasma proteome on heart failure. Front Cardiovasc Med.

[49] Sama IE, Woolley RJ, Nauta JF, Romaine SPR, Tromp J, Ter Maaten JM (2020). A network analysis to identify pathophysiological pathways distinguishing ischaemic from non-ischaemic heart failure. Eur J Heart Fail.

[50] Guo Y, Yan B, Tai S, Zhou S, Zheng XL (2021). PCSK9: Associated with cardiac diseases and their risk factors?. Arch Biochem Biophys.

[51] Xu Q, Zhao YM, He NQ, Gao R, Xu WX, Zhuo XJ (2023). PCSK9: a emerging participant in heart failure. Biomed Pharmacother.

[52] Weiner SD, Rabbani LE (2010). Secondary prevention strategies for coronary heart disease. J Thromb Thrombolysis.

[53] Jensen JS, Weeke PE, Bang LE, Høfsten DE, Ripa MS, Schjerning AM (2019). Clinical characteristics and lipid lowering treatment of patients initiated on proprotein convertase subtilisin-kexin type 9 inhibitors: a nationwide cohort study. BMJ Open.

[54] Schluter KD, Wolf A, Weber M, Schreckenberg R, Schulz R (2017). Oxidized low-density lipoprotein (oxLDL) affects load-free cell shortening of cardiomyocytes in a proprotein convertase subtilisin/kexin 9 (PCSK9)-dependent way. Basic Res Cardiol.

[55] Ding Z, Wang X, Liu S, Shahanawaz J, Theus S, Fan Y (2018). PCSK9 expression in the ischaemic heart and its relationship to infarct size, cardiac function, and development of autophagy. Cardiovasc Res.

[56] Huang G, Lu X, Zhou H, Li R, Huang Q, Xiong X (2022). PCSK9 inhibition protects against myocardial ischemia-reperfusion injury via suppressing autophagy. Microvasc Res.

[57] Palee S, McSweeney CM, Maneechote C, Moisescu DM, Jaiwongkam T, Kerdphoo S (2019). PCSK9 inhibitor improves cardiac function and reduces infarct size in rats with ischaemia/reperfusion injury: benefits beyond lipid-lowering effects. J Cell Mol Med.

[58] Yang C-L, Zeng Y-D, Hu Z-X, Liang H (2020). PCSK9 promotes the secretion of pro-inflammatory cytokines by macrophages to aggravate H/R-induced cardiomyocyte injury via activating NF-κB signalling. Gen Physiol Biophys.

[59] Wang X, Li X, Liu S, Brickell AN, Zhang J, Wu Z (2020). PCSK9 regulates pyroptosis via mtDNA damage in chronic myocardial ischemia. Basic Res Cardiol.

[60] Amput P, Palee S, Arunsak B, Pratchayasakul W, Kerdphoo S, Jaiwongkam T (2020). PCSK9 inhibitor effectively attenuates cardiometabolic impairment in obese-insulin resistant rats. Eur J Pharmacol.

[61] Lu X, Huang G, Bao H, Duan Z, Li C, Lin M (2023). Effect on hypoxia/reoxygenation-induced cardiomyocyte injury and Pink1/Parkin pathway. Gen Physiol Biophys.

[62] Wu C, Lin D, Ji J, Jiang Y, Jiang F, Wang Y (2023). PCSK9 inhibition regulates Infarction-Induced Cardiac Myofibroblast Transdifferentiation via Notch1 signaling. Cell Biochem Biophys.

[63] Wolf A, Kutsche HS, Schreckenberg R, Weber M, Li L, Rohrbach S (2020). Autocrine effects of PCSK9 on cardiomyocytes. Basic Res Cardiol.

[64] Da Dalt L, Castiglioni L, Baragetti A, Audano M, Svecla M, Bonacina F (2021). PCSK9 deficiency rewires heart metabolism and drives heart failure with preserved ejection fraction. Eur Heart J.

[65] Räber L, Ueki Y, Otsuka T, Losdat S, Häner JD, Lonborg J (2022). Effect of Alirocumab added to high-intensity statin therapy on coronary atherosclerosis in patients with Acute myocardial infarction: the PACMAN-AMI Randomized Clinical Trial. JAMA.

[66] O’Donoghue ML, Giugliano RP, Wiviott SD, Atar D, Keech A, Kuder JF (2022). Long-term evolocumab in patients with established atherosclerotic Cardiovascular Disease. Circulation.

[67] Bayes-Genis A, Lupon J, Revuelta-Lopez E, Llibre C, Gastelurrutia P, Domingo M et al. Evolocumab has no effects on heart failure with reduced ejection fraction injury biomarkers: the EVO-HF trial. Eur J Heart Fail. 2023. 10.1002/ejhf.2932 37323111

[68] Katz DH, Robbins JM, Deng S, Tahir UA, Bick AG, Pampana A (2022). Proteomic profiling platforms head to head: leveraging genetics and clinical traits to compare aptamer- and antibody-based methods. Sci Adv.

[69] Eldjarn GH, Ferkingstad E, Lund SH, Helgason H, Magnusson OT, Gunnarsdottir K (2023). Large-scale plasma proteomics comparisons through genetics and disease associations. Nature.

