# Supplementary material for: Proteome-wide mendelian randomization investigates potential associations in heart failure and its etiology: emphasis on PCSK9
Source: BMC Med Genomics. 2024 Feb 21;17:59. doi: 10.1186/s12920-024-01826-6 (PMC10882912; doi:10.1186/s12920-024-01826-6)
Supplement: Supplementary file 1 — Supplementary Material 1 [file 12920_2024_1826_MOESM1_ESM.docx]

**Supplementary figures**


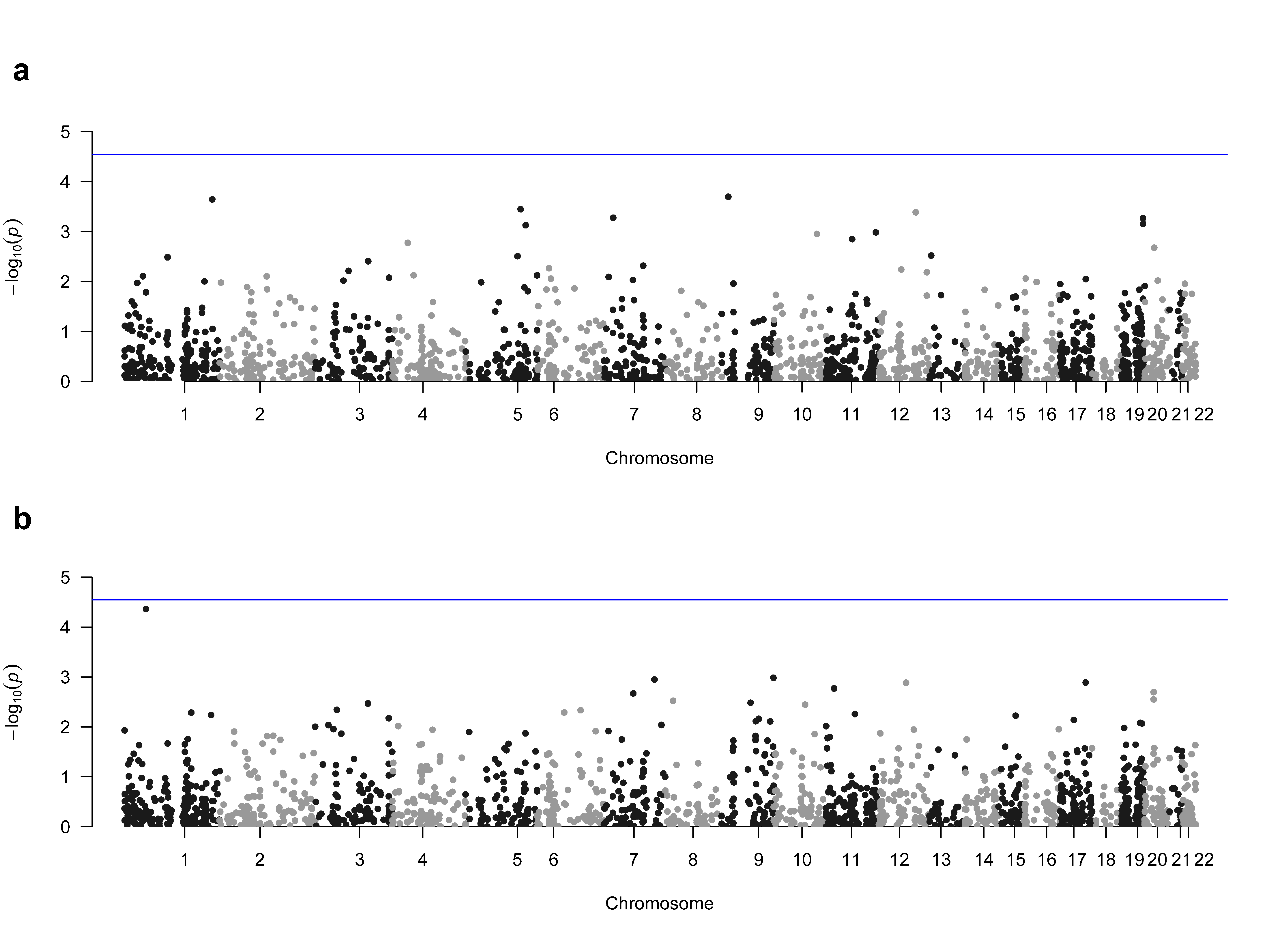


**Fig. S1** Manhattan plots for associations of genetically predicted circulating proteins levels with all-cause HF in MR analysis. a) Associations of genetically predicted circulating proteins with all-cause HF in the HERMES Consortium; b) Associations of genetically predicted circulating proteins with all-cause HF in the FinnGen Consortium. Blue line indicates the Bonferroni-corrected P = 0.05. Results are plotted by gene start position.

**
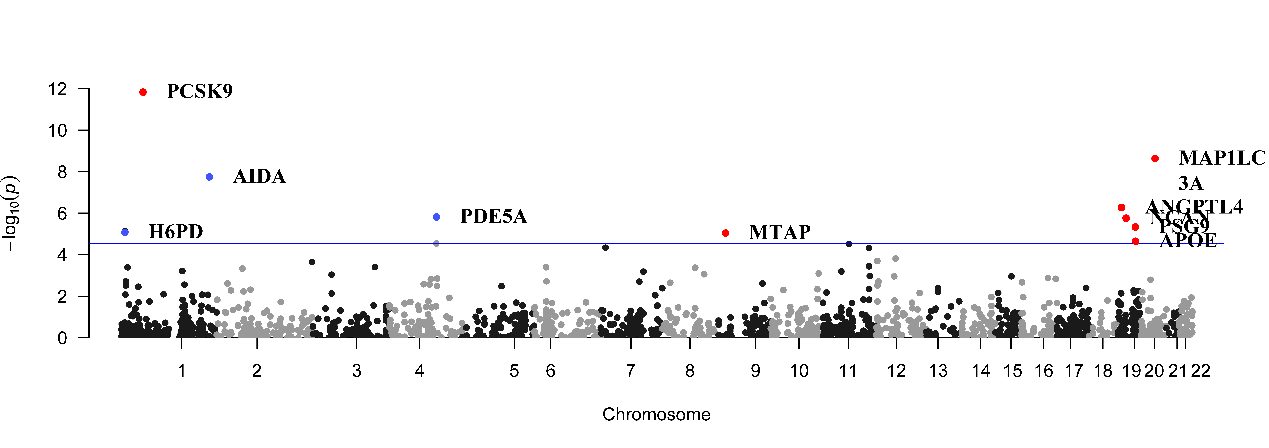
**

**Fig. S2** Manhattan plots for associations of genetically predicted circulating proteins levels with ischemic heart disease in MR analysis. Associations of genetically predicted circulating proteins with ischemic heart disease. Labelled and colored proteins refer to MR findings with Bonferroni-corrected P < 0.05 (two-sample MR analysis). Red proteins indicate the positive effect of the circulating proteins on outcomes; blue proteins indicate the negative effect of the circulating proteins on outcomes; blue line indicates the Bonferroni-corrected P = 0.05. Results are plotted by gene start position.


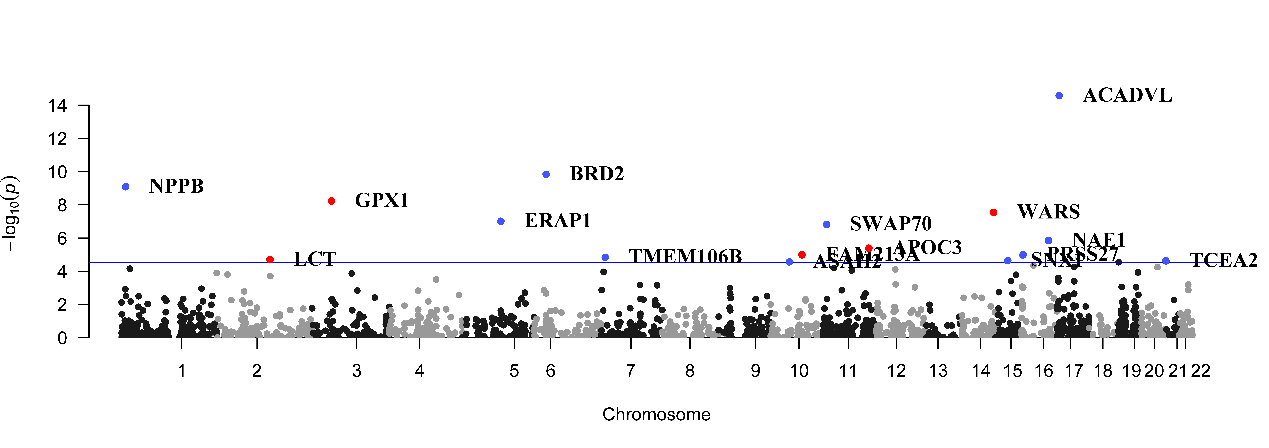


**Fig. S3** Manhattan plots for associations of genetically predicted circulating proteins levels with essential hypertension in MR analysis. Associations of genetically predicted circulating proteins with essential hypertension. Labelled and colored proteins refer to MR findings with Bonferroni-corrected P < 0.05 (two-sample MR analysis). Red proteins indicate the positive effect of the circulating proteins on outcomes; blue proteins indicate the negative effect of the circulating proteins on outcomes; blue line indicates the Bonferroni-corrected P = 0.05. Results are plotted by gene start position.

**
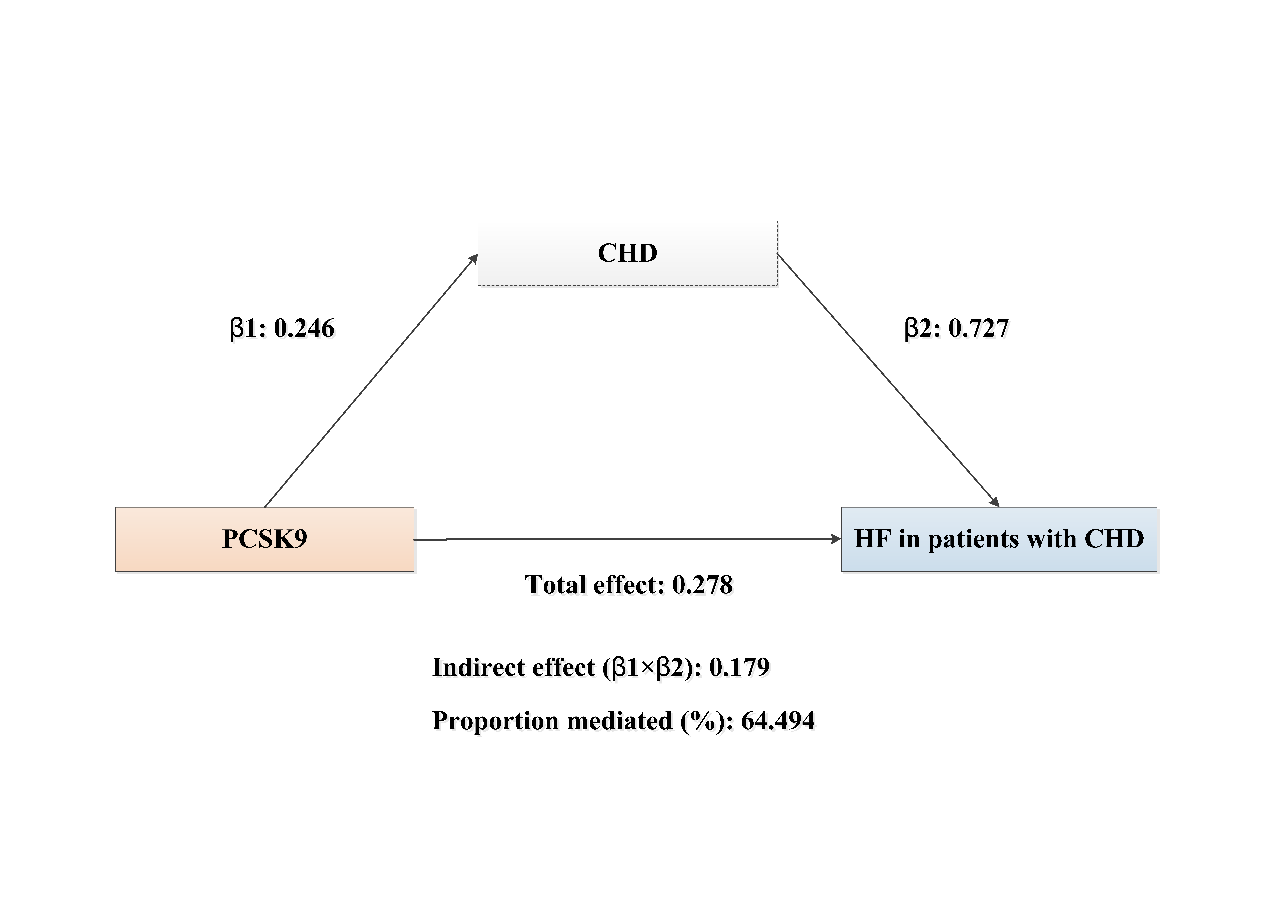
**

**Fig. S4** Two-Step Mendelian randomization analysis assessing the mediation effect of CHD on the relationship between PCSK9 and HF in patients with CHD. HF: heart failure; CHD: Coronary heart disease; β1 = Beta value between PCSK9 and mediator; β2 = Beta value between mediator and HF in patients with CHD; Total effect = Beta value between PCSK9 and HF in patients with CHD; Proportion of mediation (%) = (Indirect effect / Total effect) × 100%.


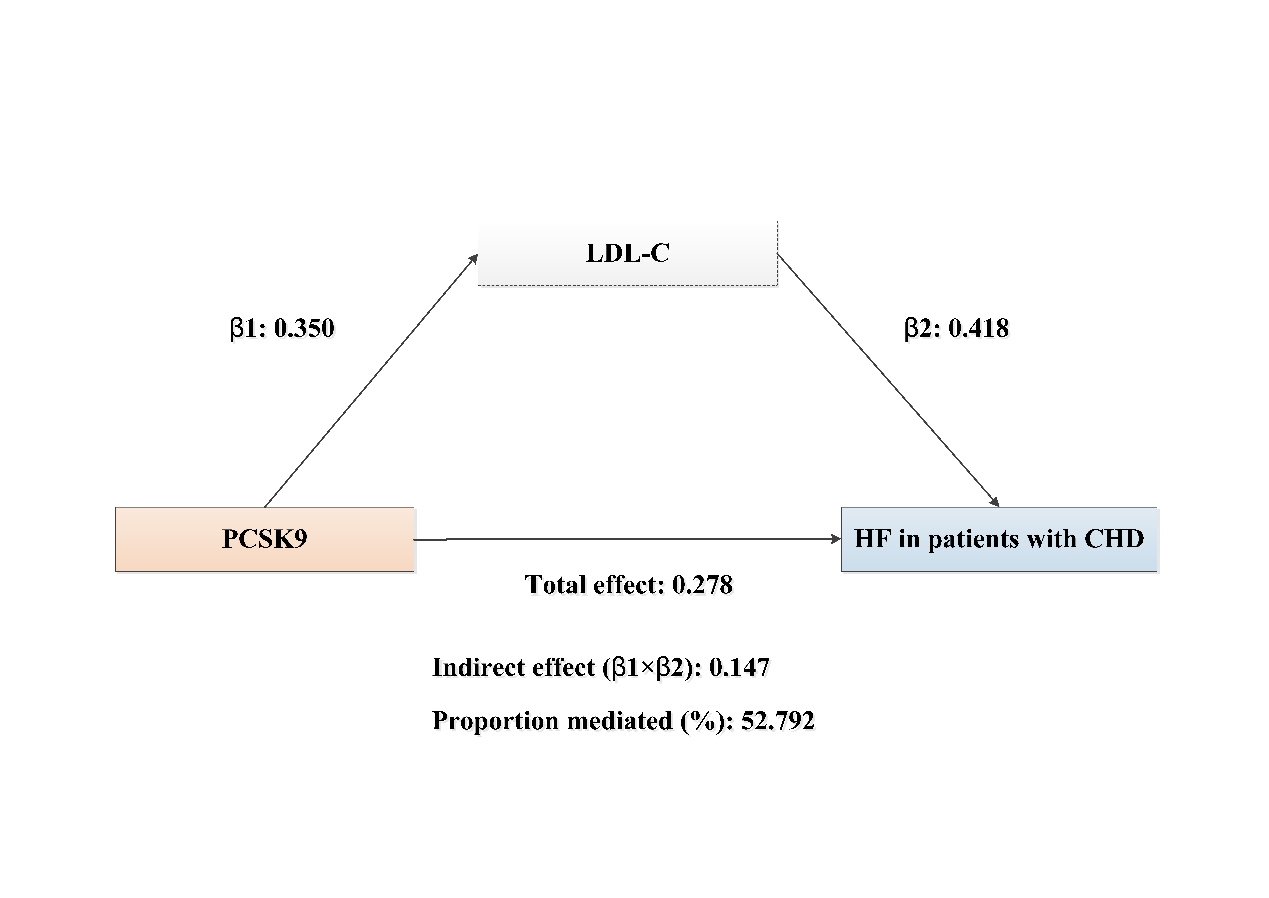


**Fig. S5** Two-Step Mendelian randomization analysis assessing the mediation effect of LDL-C on the relationship between PCSK9 and HF in patients with CHD. HF: heart failure; CHD: Coronary heart disease; LDL-C: low-density lipoprotein; β1 = Beta value between PCSK9 and mediator; β2 = Beta value between mediator and HF in patients with CHD; Total effect = Beta value between PCSK9 and HF in patients with CHD; Proportion of mediation (%) = (Indirect effect / Total effect) × 100%.


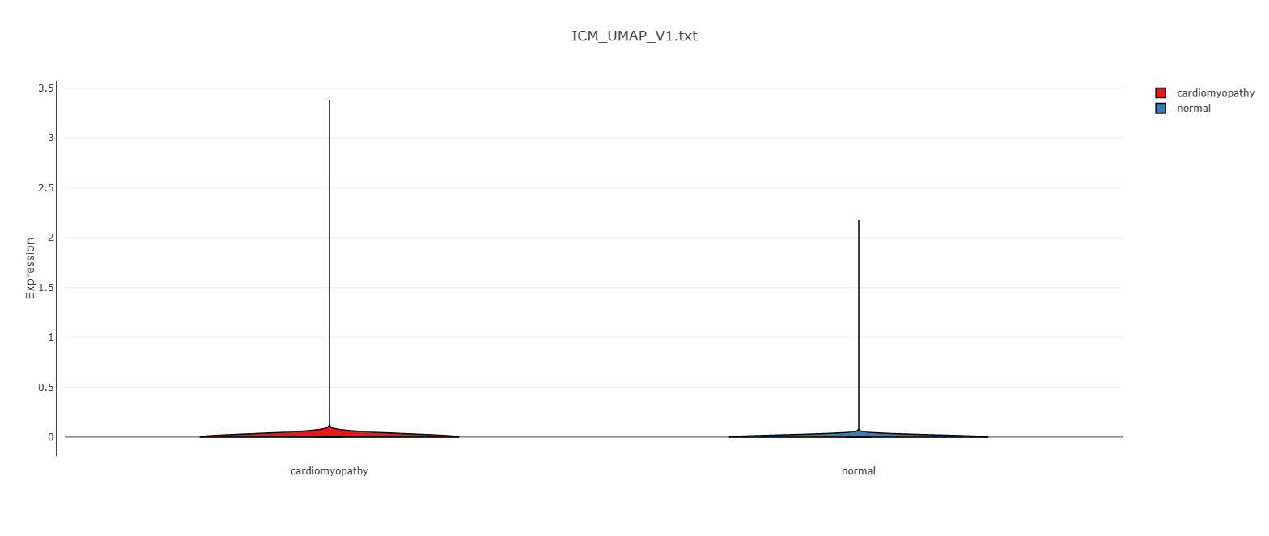


**Fig. S6** Comparing PCSK9 expression in cardiac cells between End-Stage Ischemic Cardiomyopathy and Non-Failing Controls. In this figure, the red group correspond to ischemic cardiomyopathy at end-stage HF, while the blue group represent the non-failing controls. The vertical axis denotes the PCSK9 expression levels.


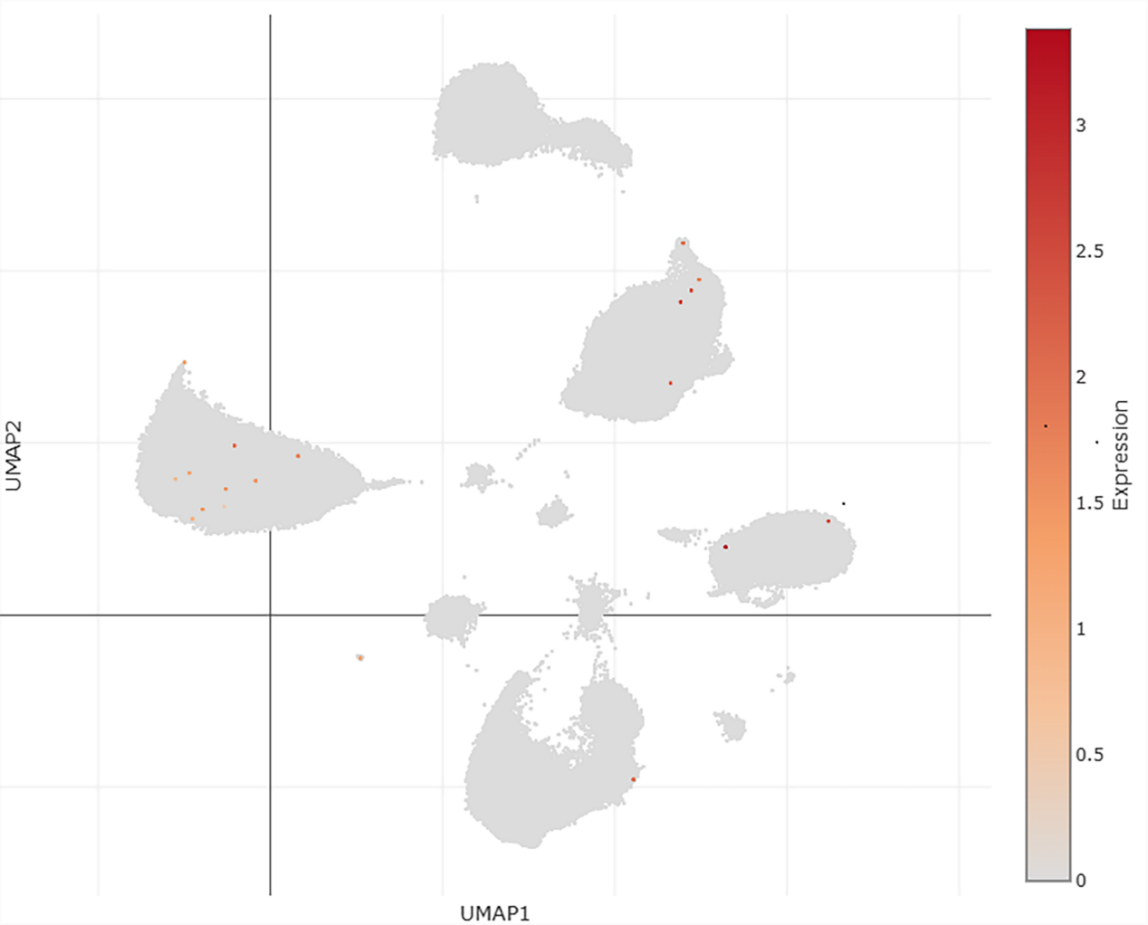


**Fig. S7** Expression of PCSK9 in cardiac cells in both End-Stage Ischemic Cardiomyopathy and Non-Failing Controls. In the UMAP dimensionality reduction cluster diagram, each data point corresponds to an individual cell. Intensity of color indicates the relative expression level of PCSK9 within the cell, with redder shades denoting higher expression.
